# Comparison Between Pressure Swing Adsorption and Liquid Oxygen Enrichment Techniques in the Atacama Large Millimeter/Submillimeter Array Facility at the Chajnantor Plateau (5,050 m)

**DOI:** 10.3389/fphys.2021.775240

**Published:** 2021-12-08

**Authors:** Ivan Lopez, Reinaldo Aravena, Daniel Soza, Alicia Morales, Silvia Riquelme, Rodrigo Calderon-Jofré, Fernando A. Moraga

**Affiliations:** ^1^Atacama Large Millimeter/Submillimeter Array, San Pedro de Atacama, Chile; ^2^Departamento de Medicina, Facultad de Ciencias de la Salud, Universidad de Tarapacá, Arica, Chile; ^3^Laboratorio de Fisiología, Hipoxia y Función Vascular, Departamento de Ciencias Biomédicas, Facultad de Medicina, Universidad Católica del Norte, Coquimbo, Chile

**Keywords:** pressure swing adsorption (PSA), liquid oxygen, oxygen generation, operation at 5050 m, heart rate, oxygen saturation (SpO_2_), portable oxygen, chronic intermittent hypobaric hypoxia

## Abstract

The Chilean workforce has over 200,000 people that are intermittently exposed to altitudes over 4,000 m. In 2012, the Ministry of Health provided a technical guide for high-altitude workers that included a series of actions to mitigate the effects of hypoxia. Previous studies have shown the positive effect of oxygen enrichment at high altitudes. The Atacama Large Millimeter/submillimeter Array (ALMA) radiotelescope operates at 5,050 m [Array Operations Site (AOS)] and is the only place in the world where pressure swing adsorption (PSA) and liquid oxygen technologies have been installed at a large scale. These technologies reduce the equivalent altitude by increasing oxygen availability. This study aims to perform a retrospective comparison between the use of both technologies during operation in ALMA at 5,050 m. In each condition, variables such as oxygen (O_2_), temperature, and humidity were continuously recorded in each AOS rooms, and cardiorespiratory variables were registered. In addition, we compared portable O_2_ by using continuous or demand flow during outdoor activities at very high altitudes. The outcomes showed no differences between production procedures (PSA or liquid oxygen) in regulating oxygen availability at AOS facilities. As a result, big-scale installations have difficulties reaching the appropriate O_2_ concentration due to leaks in high mobility areas. In addition, the PSA plant requires adequacy and maintenance to operate at a very high altitude. A continuous flow of 2–3 l/min of portable O_2_ is recommended at 5,050 m.

## Introduction

High altitude is a geographical condition where barometric pressure and inspired partial pressure of oxygen (PiO_2_) are reduced with altitude. Today, about 140 million people live or work at altitudes above 2,500 m (Moore, 2001). According to the periodicity of exposure, hypoxia can be acute (observed in tourists, climbers, and hikers), chronic (people who live permanently at high altitudes, between 3,000 and 4,500 m), and intermittent (people who alternate exposure between hypoxia and normoxia) (Gassmann et al., 2021). However, the term “intermittent hypoxia” refers to a wide spectrum of contexts: episodic intermittent hypoxia (EIH), in obstructive sleep apnea-hypopnea syndrome (OSAHS); intervallic intermittent hypoxia (IIH), during intercontinental commercial flight crews; and the chronic intermittent hypobaric hypoxia (CIHH), in people who work under a shift system at high altitude and rest at sea level (Viscor et al., 2018).

The CIHH model of exposure is common in the mining industry in the Andes and the Central Asian regions but is also observed in astronomical observatories and border control personnel (including military, police, and customs) in many high-elevation countries (Moraga et al., 2014, 2018). In Chile, over 200,000 people ascend to high altitudes for work (Consejo de Competencias Mineras, 2017, available at http://www.ccm.cl/ReporteCCM_13-11_FINAL). Nowadays, we know that this exposure model is associated with acute mountain sickness, sleep disorders, polycythemia, pulmonary hypertension, and an acute increase in arterial pressure (Ministry of Health Chile, 2014, available at https://www.minsal.cl/sites/default/files/guia_hipobaria_altitud.pdf). These symptoms are present each time workers arrive at their high altitude worksite and do not disappear over time (Richalet et al., 2002; Moraga et al., 2014, 2018). To meet the requirements of the work unions of mining companies and studies about the safety of work at high altitudes, the Chilean Ministry of Health recognized altitude exposure as a health risk and by Decree 28 of 2013 defined chronic intermittent hypoxia exposure when workers are exposed to altitudes over 3,000 m for business reasons for more than 6 months, with a minimum stay of 30% of the time in rotating work shifts at high altitude and rest at low altitude (Ministry of Health Chile, 2013, available at http://bcn.cl/1vgrd). Considering this, in 2014, the Ministry of Health provided a technical guide for high altitude workers that includes a series of recommendations to reduce malaise or risk during exposure to altitudes over 3,000 m and considers altitudes over 5,500 m as extreme altitudes. In regard to environmental oxygenation, the oxygen equivalent must be below 3,000 m with permanent control of temperature, relative humidity, and room ventilation (Ministry of Health Chile, 2014, available at https://www.minsal.cl/sites/default/files/guia_hipobaria_altitud.pdf).

One way to avoid the consequences of high-altitude hypoxemia is to reduce the equivalent altitude (altitude that provides the same PiO_2_ when ambient air with an oxygen concentration of 21% is inhaled) (West, 2002). The first physiological response is increased pulmonary ventilation, which can be reduced by increasing the room PiO_2_ with an artificial increase in oxygen supply (Cudaback, 1984). West (2016) explored the possibility of oxygen supplementation at high altitudes to reduce the malaise associated with high altitude exposure, i.e., miners camps, observatories, hotels, trains, schools, and hospitals. He defined that each increase of oxygen concentration by 1% resulted in a reduction equivalent to 300 m (West, 1995, 2002; Luks et al., 1998). In an oxygen-enriched ambient air room, the oxygen is a comburent and significantly increases the risk of ignition and fire. However, the fire hazard in ambient air decreases at higher altitudes than sea level since the decrease in oxygen partial pressure reduces the number of oxygen molecules available for combustion (West, 1995, 2015). Thus, despite oxygen enrichment of the atmosphere at high altitudes, the PiO_2_ is still far below that of air at sea level.

Nowadays, it is possible to recognize two different procedures to reduce the equivalent altitude by oxygen enrichment in the air ambient of a workplace at high altitudes (i.e., room dormitories, office): First, an oxygen concentrator, a device that increases oxygen concentration to 90–95%, through a separation process that employs a technology called pressure swing adsorption (PSA). In this system, a non-flammable ceramic material (zeolite) adsorbs N_2_ more readily than O_2_, increasing oxygen concentration in the delivered gas mixture (West, 1995). In a series of studies, oxygen concentrators were considered as low-cost systems in installation and maintenance and only required electrical power (West, 1995, 2002; Luks et al., 1998). Second, the use of liquid oxygen, which requires the installation of bigger tanks with liquid oxygen obtained from a cryogenic plant. In both the cases (oxygen concentrators or liquid oxygen), it is required to install pipelines, valves, sensors, and control systems to maintain O_2_, CO_2_, temperature, and humidity (West, 1995, 2002; Moraga et al., 2014, 2018).

The Atacama Large Millimeter/submillimeter Array (ALMA) is a radiotelescope operating at 5,050 m in the Chajnantor Plateau. The ALMA operation is carried out by near of 400 workers and has two locations: the first is the base camp or Operations Support Facility (OSF), located 16 km from the town of San Pedro de Atacama and situated at 2,900 m, providing all the personnel facilities (residential, food services, and leisure facilities) and the second is the Array Operations Site (AOS), located 30 km from the OSF at an altitude of 5,050 m. The ALMA is the only place in the world where both the technologies (PSA and liquid oxygen) have been installed at a large scale to reduce equivalent altitude by increasing oxygen availability at very high altitudes. In this way, the aim of this study is a retrospective comparison between the use of both technologies for oxygen supplementation during the operation of the ALMA laborers at 5,050 m.

## Subjects, Materials, and Methods

All the evaluated workers lived at a low altitude (<1,000 m) and worked at the ALMA (2,900 and 5,050 m), with a shift pattern of 8 days of work at high altitude followed by 6 days of rest at sea level. All the subjects worked as operators and maintenance crew with CIHH exposure experience for more than 4 years and were free of cardiovascular, pulmonary, hematological, renal, or hepatic diseases. This study complied with the Helsinki guidelines and was previously approved by the Ethics Committee of the Facultad de Medicina of Universidad Catoìlica del Norte and the ALMA Safety Department.

### Oxygen Enrichment Inside the AOS Building Areas at 5,050 m

**FIGURE 1:**
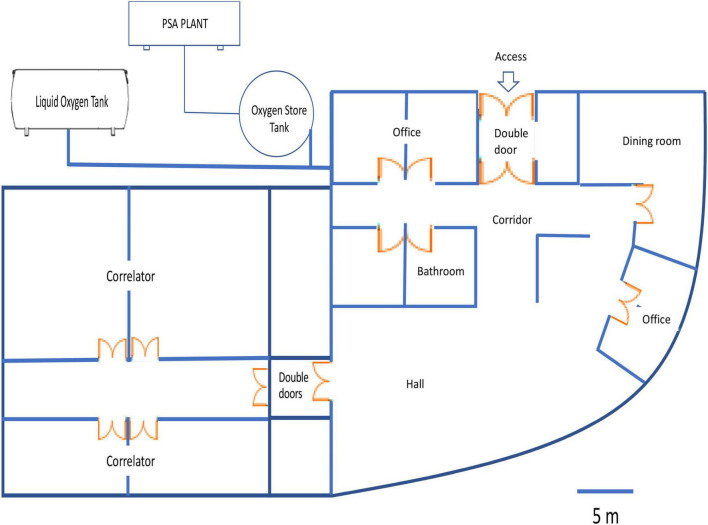
The scheme represents a diagram of oxygen enrichment inside Array Operations Site (AOS) facilities at 5,050 m.

In 2008, the ALMA installed an onsite oxygen generator machine capable of producing oxygen by using PSA technology to satisfy oxygenation requirements in all the AOS rooms at 5,050 m, obtaining an oxygen concentration of 28%, equivalent to an altitude of 2,900 m. The installed oxygen system has two external tanks (2,498 L × 2). The maximum oxygen pressure is 75 psi with a delivery of 850 l/min with 90–95% oxygen purity and 45 psi outlet pressure. The oxygen concentration in the ambient air was controlled by a wide range oxygen sensor (0–50%, accuracy ± 0.05%, Area Safety Monitor, model 221, Advanced Micro Instruments, Costa Mesa, CA, United States) and maintained by a precise servo control system that increased oxygen concentration to 28 ± 0.5%. All the ambient variables (O_2_, temperature, and humidity) were recorded continuously in the available AOS rooms (corridor, hall, office, and correlator; Figure 1). All the variables recorded, such as O_2_, temperature, and humidity, were compiled monthly for at least 1 year for each AOS room. After installing and operating the PSA, we performed cardiorespiratory evaluations in 9 of 13 acclimatized AOS workers of 30.5 ± 6.4 years. A nurse or paramedic measured variables such as heart rate (HR) (bpm) and pulse oxygen saturation (SpO_2_) (%) using a multiparameter monitor (model BM3, Bionet, Tustin, CA, United States). However, in 2011, the ALMA shut down the oxygen administration procedure in the building areas at 5,050 m due to problems in the operation of the PSA plant.

In 2015, one liquid oxygen tank was installed with a maximal net volume of 14.75 m^3^, enabling a 70 normalized m^3^/h (or Nm^3^/h) flow rate (installation and liquid oxygen supply was performed by Indura). The ambient oxygen concentration, temperature, and humidity were controlled and maintained, as previously described. Additionally, we evaluated cardiorespiratory parameters routinely at the arrival of 40 workers to AOS rooms and values represent the mean of 1 year of operation. In this case, the evaluation of variables such as HR (bpm) and SpO_2_ (%) was measured by a nurse or paramedic using a multiparameter monitor (model BM3, Bionet, Tustin, CA, United States).

### Use of Portable Oxygen in Outdoor Operations at 5,050 m

We evaluated efficiency during a period of workers’ activity (changing an antenna). We defined the oxygen flow requirements using two different pieces of equipment: first is a piece of equipment that supplies oxygen by continuous flow (range flow of 0.5–15 l/min, model 108 MF 870, Corpus™ INDURA, Santiago, Chile) and the second the CHAD^®^ Evolution™ Motion Auto-Adjusting Oxygen Conserver (probably model OM-900M; Drive DeVilbiss Healthcare, Somerset, PA, United States), both gaseous medicinal oxygen, compressed in the high-pressure cylinder, were administrated *via* a nasal cannula. We performed dose-response curves of oxygen saturation vs. flow breathing between 0 and 6 l/min in six antenna maintenance personnel. We continuously recorded the subjects while changing an antenna with a pulse oximeter (Wristox 3100, Nonin, MN, United States). All the recordings were analyzed by nVision software (Nonin, MN, United States).

### Statistical Analysis

All the results were expressed as mean ± SD. Variables compiled of AOS rooms O_2_, temperature, and humidity of PSA or liquid oxygen were analyzed using the paired *t*-test. Cardiorespiratory variables SpO_2_ and HR recorded in AOS rooms or taken outdoors were analyzed using the paired *t*-test. All the differences were considered statistically significant when *p* < 0.05. Data analyses were performed using the GraphPad Prism version 5.03 (GraphPad Software Incorporation, San Diego, CA, United States).

## Results

### Oxygen Enrichment Inside Array Operations Site Facilities at 5,050 m

A similar oxygenation pattern was observed in both the procedures to increase oxygen concentration (PSA or liquid oxygen) inside AOS rooms. Table 1 shows ambient measurements of O_2_, relative humidity, and temperature at all the AOS sites without an observed difference. O_2_ variations in the corridor, hall room, and office were not significantly different between the PSA and liquid oxygen system 26.5 ± 1.2 and 26.1 ± 1.3%, respectively. Furthermore, when evaluating cardiorespiratory responses such as oxygen saturation and HR in all the subjects during ascents to AOS, subjects presented increased arterial oxygenation and reduction in the HR in both the conditions (PSA and liquid oxygen) (Table 2).

**TABLE 1:** Conditioning and ambient oxygen (O_2_) enrichment in the Array Operations Site (AOS) building areas.

	O_2_ enrichment in rooms (%)	
	PSA	Liquid oxygen
**Areas in AOS**		
Corridor	26.5 ± 1.0	25.5 ± 1.5
Hall	26.5 ± 1.4	26.3 ± 1.3
Dining room	-	26.5 ± 1.2
Office	26.6 ± 1.3	27.5 ± 0.8
Correlator	27.8 ± 0.5	28.0 ± 0.5
All AOS	26.8 ± 1.1	26.8 ± 1.1
Temperature (°C)	17.5 ± 1.5	18.5 ± 1.2
Relative Humidity (%)	33.2 ± 7.5	29.1 ± 3.1

*Mean ± SD.*

**TABLE 2:** Comparison of cardiorespiratory variables in O_2_-enriched AOS rooms.

	Arrive at 5050 m	
	(Outside AOS)	(Inside AOS + O_2_)
**PSA**		
Oxygen saturation (%)	83.1 ± 2.3	92.8 ± 1.2*
Heart rate (bpm)	102 ± 13	93.1 ± 10.6
**Liquid oxygen**		
Oxygen saturation (%)	82.6 ± 2.8	89.5 ± 1.4*
Heart rate (bpm)	110.0 ± 9.2	78.6 ± 8.6*

Mean ± SD, *p < 0.05 outside vs. inside.

### Use of Portable Oxygen in Outdoor Operations

**FIGURE 2:**
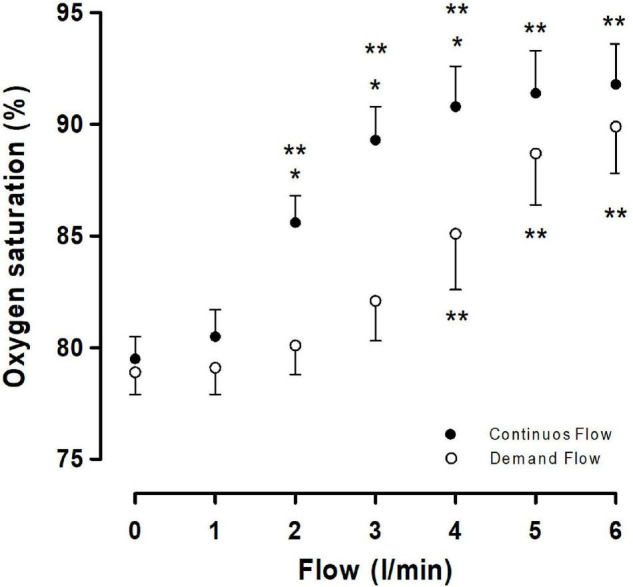
Oxygen saturation dose-response curve vs. inhalatory flow (l/min). Closed circle represents the use of a continuous flow and open circle represents the use of a demand flow. Each circle represents a mean ± SD. *p < 0.05 continuous vs. demand and **p < 0.05 vs. 0 flow.

The use of individual oxygen supplementation showed that efficient oxygenation, equivalent to 2,900 m, was obtained using a continuous flow (3 l/min) rather than the demand flow that obtained equivalent values at 5 l/min (Figure 2).

## Discussion

### Oxygen Enrichment Within Array Operations Site Facilities at 5,050 m

Our results demonstrate that both the oxygen production procedures (PSA or liquid oxygen) effectively increase the fraction of inspired oxygen (FiO_2_) at very high altitudes, protecting the health of the workers as noted by the increase in oxygen saturation and reduced HR.

The use of an oxygen concentrator (PSA plant) vs. liquid oxygen for oxygen enrichment produced similar oxygenation patterns at AOS facilities at 5,050 m. These patterns were characterized by a variation in the oxygen concentration within AOS facilities. Even though these facilities have double door access, these oxygen variations could be explained by the movement of personnel, resulting in the doors being constantly opened and closed and promoting a fall in FiO_2_ to 26.5%. In contrast, offices and correlators have access through doors that are usually maintained shut. In this pilot study, we used a comfortable and mobile module in the AOS facilities at 5,050 m where liquid oxygen was administered to the room air to increase oxygen concentration to 28 ± 0.5%. This oxygen concentration represents an equivalent altitude of 2,900 m (for more detail, see Moraga et al., 2018), with higher precision and maintenance of the oxygen concentration, potentially explained by a reduced module size where the leakage is lower. However, when we measured cardiorespiratory response before or after access to oxygenated AOS facilities, a similar increase in oxygen saturation was observed in both the oxygenation procedures. In contrast, we only observed a reduced HR in workers within AOS facilities with liquid oxygen, similar to previous reports (Moraga et al., 2018).

The PSA plant oxygen concentrator was shut down, even though a series of studies emphasize that oxygen concentrators are low cost in installation and maintenance and only require electrical power support (West, 1995, 2002; Luks et al., 1998). It was also proposed that the molecular sieve will last indefinitely (West, 1995, 2015), as long as it does not become contaminated with water and oil vapors (Litch and Bishop, 2000). However, after installation in 2008, a series of operational troubles were reported such as not reaching the required oxygenation. Inspection of the PSA plant following shutdown reported a series of malfunctions, starting with oil contamination along the air preparation route. This fact was unacceptable due to the risk of rapid self-ignition and possible explosions with fatal consequences for the ALMA operating staff. In addition, zeolite beds, O_2_ storage tanks, and pipelines needed to be exchanged due to the oil and water contamination mentioned in the same report. This experience was corroborated in an article that mentioned regular maintenance is required such as cleaning cabinet filters, changing bacterial and particulate filters, and zeolite columns after 15,000–20,000 h of use, depending on local conditions (Litch and Bishop, 2000). The same authors indicated that output oxygen concentration at high altitudes decreases by approximately 10% for each 2,000 m gain in elevation (Litch and Bishop, 2000). Therefore, we expect a decrease in oxygen concentration by 25% at 5,050 m. This suggests that the PSA plant at very high altitude its efficiency decreases. Then, how is it possible that this technology is not helpful at very high altitudes? In our opinion, the studies that supported this technology did not consider the loss in equipment efficiency at very high altitudes, as mentioned by Litch and Bishop (2000). In this sense, we believe that installing the PSA plant at high and very high altitudes requires that the efficiency of this technology is validated in real ambient conditions such as very low pressure, dust, and air dry and consider periodic maintenance.

Previous studies developed in real conditions at 4,200 m used liquid oxygen to enhance sleep quality (Moraga et al., 2014). In the Collahuasi mine, the same solution was taken at a large scale with 60 oxygen-enriched dormitory rooms using liquid oxygen at 3,800 m (West, 2004). However, we recognize the major weakness of this procedure for oxygen-enrichment areas given by the complications associated with refilling liquid oxygen tanks due to road accessibility and weather conditions. However, programming delivery, having backup tanks, and providing supplemental oxygen equipment could cover any delays.

### Use of Portable Oxygen in Outdoor Operations

Our results show that oxygen administration was more efficient when using a continuous flow than a demand flow. This was proven by arterial oxygen saturation, where values of 2–3 l/min for continuous flow reached saturation values over 85%, but 5 l/min was necessary with the demand flow. A similar pattern was observed in a study showing that the administration of low-flow oxygen (1 or 3 l/min) *via* a face mask enhances physical performance in unacclimatized subjects following rapid ascent to the ALMA in the Chajnantor plateau (Silva-Urra et al., 2011). However, previous studies reported oxygen saturation values lower than expected at 5,050 m (Moraga et al., 2018), suggesting that a flow of 1 or 3 l/min was insufficient. An explanation based on the results reported by Silva-Urra et al. (2011) is that the study was performed in acutely exposed subjects to 5,050 m. In contrast, our results were obtained in acclimatized personal evaluated in AOS. The outcomes of this study are that all the subjects that ascend (visitors and operators) to AOS and outdoor activities and drivers of light or heavy trucks must use oxygen with a continuous O_2_ flow of 2.0–3.0 l/min to maintain an O_2_ saturation of 85–91%, supporting that the demand flow is not recommended at very high altitude.

## Conclusion

No differences were observed between both the oxygen production procedures (PSA or liquid oxygen) in regular operations. Big-scale installations have difficulties reaching the oxygen concentration due to leaks in areas of high mobility. However, the PSA plant requires adequacy and maintenance to operate at a very high altitude. At 5,050 m, portable oxygen should operate with a continuous flow of 2–3 l/min. In addition, new equipment for individual oxygen supplementation at 5,050 m must be evaluated, considering ergonomics and autonomy during exposure/operation.

## Data Availability Statement

The raw data supporting the conclusions of this article will be made available by the authors, without undue reservation.

## Ethics Statement

The studies involving human participants were reviewed and approved by Ethics Committee of the Facultad de Medicina of Universidad Católica del Norte. The patients/participants provided their written informed consent to participate in this study.

## Author Contributions

IL and FM were the guarantors and conceived and designed the study. RA, SR, and AM supervised the overall study. RC-J and DS contributed to sample and data collections. RC-J performed the statistical analysis. All authors drafted the report, interpreted the results, critically revised the manuscript, and approved the final manuscript.

## Conflict of Interest

The authors declare that the research was conducted in the absence of any commercial or financial relationships that could be construed as a potential conflict of interest.

## Publisher’s Note

All claims expressed in this article are solely those of the authors and do not necessarily represent those of their affiliated organizations, or those of the publisher, the editors and the reviewers. Any product that may be evaluated in this article, or claim that may be made by its manufacturer, is not guaranteed or endorsed by the publisher.
